# A Cross‐Climate Comparison of Molecular Phenology in Three Tropical and Temperate Trees

**DOI:** 10.1002/pei3.70146

**Published:** 2026-04-09

**Authors:** Atsuko Miyawaki‐Kuwakado, Nakata Taichi, Yuka Ikezaki, Naoki Tani, Yoshiko Kosugi, Kevin Kit Siong Ng, Soon Leong Lee, Akiko Satake

**Affiliations:** ^1^ Department of Biology, Faculty of Science Kyushu University Fukuoka Japan; ^2^ Forestry Division Japan International Research Center for Agricultural Sciences Tsukuba Ibaraki Japan; ^3^ Institute of Life and Environmental Sciences University of Tsukuba Tsukuba Ibaraki Japan; ^4^ Graduate School of Agriculture, Kyoto University Kyoto Japan; ^5^ Forestry Biotechnology Division Forest Research Institute Malaysia Kepong Selangor Malaysia

**Keywords:** forest ecosystems, gene expression, latitudinal gradients, photosynthesis, stress response, temperature fluctuation, transcriptomics

## Abstract

Latitudinal gradients in temperature seasonality shape the evolution of thermal tolerance and acclimation capacity in organisms. According to Janzen's climate variability hypothesis, tropical species experiencing stable temperatures evolve narrower thermal niches than temperate counterparts. To test whether this principle extends to gene expression dynamics, we compared annual transcriptome profiles of the tropical tree *Rubroshorea leprosula* and the temperate evergreen trees *Lithocarpus edulis* and *Quercus glauca* under natural field conditions. Time‐series RNA‐seq analyses revealed that *R. leprosula* exhibited sporadic transcriptional shifts triggered by slight cooling events (minimum temperatures of 21°C–22°C), whereas the temperate species showed clear annual cycles characterized by winter‐specific expression patterns. Mild temperature decline in the tropical tree triggered widespread down‐regulation of photosynthesis‐related genes and activation of stress‐response and jasmonate‐associated signaling pathways, suggesting coordinated responses even to mild temperature declines. Cross‐species comparison of 3793 single‐copy orthologs showed that the sensitivity to temperature and dynamic range of gene expression were substantially larger relative to the narrow dynamic range of temperature in the tropics, indicating amplified transcriptional responses per unit of temperature variation. Conversely, temperate species displayed broad but proportionate transcriptomic shifts that paralleled large seasonal temperature fluctuations. These results demonstrate that transcriptional sensitivity to temperature is exaggerated in tropical species and buffered in temperate ones, extending Janzen's climate variability hypothesis from physiological tolerance to its molecular basis.

## 1 Introduction

The survival, growth, and reproduction of living organisms are influenced by environmental factors, including temperature, solar radiation, and humidity. Global cooling during the Cenozoic established strong latitudinal gradients in temperature seasonality (Toumoulin et al. 2022). At higher latitudes, temperatures fluctuate markedly across seasons, whereas equatorial regions are characterized by relatively stable temperatures throughout the year (Satake et al. 2022). The extent of temperature variation shapes the evolution of physiological tolerance ranges in organisms. Janzen's climate variability hypothesis (Janzen 1967) posits that greater environmental temperature variation selects for broader thermal tolerance in organisms (Stevens 1989; Ghalambor et al. 2006). Building on this idea, Janzen proposed that tropical organisms, which experience relatively stable temperatures year‐round, evolve narrow thermal niches and limited acclimation capacity.

Comparative studies on thermal tolerance between tropical and temperate organisms have shown that tropical species of insects, amphibians, reptiles, and fish tend to have narrower thermal tolerance ranges defined as thermal maxima minus minima than their temperate counterparts (Deutsch et al. 2008; Sunday et al. 2010). In plants as well, the breadth of thermal tolerance, encompassing both heat and cold tolerance, is thought to have evolved in response to the degree of climatic variability in their native environments (O'Sullivan et al. 2017; Crous et al. 2022). Araújo et al. (2013) demonstrated that upper thermal limits are evolutionarily conserved across a wide range of taxa, including plants and ectothermic animals, whereas lower thermal limits are more evolutionarily labile. This evolutionary asymmetry indicates that upper thermal tolerance is constrained and evolves slowly, limiting the capacity of tropical species to adapt to rapidly warming climates. Indeed, projected extinction rates are expected to be highest for species inhabiting low‐latitude biodiversity hotspots (Malcolm et al. 2006; Román‐Palacios and Wiens 2020).

To gain a deeper understanding of the physiological basis underlying these contrasting thermal responses, it is useful to examine how organisms from tropical and temperate regions differ in their gene expression dynamics under natural environmental fluctuations. Transcriptomic analyses offer a powerful approach to uncover temperature‐sensitive molecular processes that operate well before the onset of physiological stress. By applying this approach across biogeographic regions, we can identify genes and pathways that respond most sensitively to temperature variation. We can then test whether tropical species, constrained by narrower thermal niches, exhibit reduced capacity to maintain physiological function outside their typical temperature range. In reef‐building corals, experimental studies have shown that gene expression profiles can predict thermal tolerance. For example, Barshis et al. (2013) reported that heat‐tolerant coral populations exhibit constitutively elevated expression of stress‐response genes. Seneca and Palumbi (2015) introduced the concept of transcriptome resilience—faster return to baseline expression after heat stress—and Kenkel and Matz (2016) demonstrated that transcriptional flexibility is adaptive under variable thermal conditions. Comparative transcriptomic studies between temperate and tropical populations or across distinct biomes have also been conducted in insects (Levine et al. 2011) and plants (Andrew et al. 2025). *Drosophila melanogaste* originating from tropical and temperate regions (Levine et al. 2011), as well as 20 native Australian plant species from arid, alpine, and temperate biomes (Andrew et al. 2025), have been reared or exposed to different temperature conditions, and the resulting changes in gene expression have been comprehensively analyzed. These studies have revealed differences in temperature‐responsive expression plasticity among populations and biomes, suggesting that gene expression regulation contributes to local adaptation (Rivera et al. 2021).

While these studies rely on controlled temperature or transplant experiments, transcriptional responses to naturally fluctuating environments remain poorly understood. In sessile organisms, particularly plants, transcriptomic studies are increasingly incorporating long‐term field observations under natural conditions—an approach referred to as *molecular phenology* (Kudoh 2016; Nagano et al. 2019; Dai et al. 2023; Komoto et al. 2024; Satake et al. 2023; Satake, Hagiwara, et al. 2024; Miyawaki‐Kuwakado et al. 2024; Kudo et al. 2025). Trees, in particular, provide an excellent system for investigating gene expression responses to seasonal and interannual climatic variation in ecologically realistic settings. Their long lifespan and continuous exposure to environmental fluctuations allow the detection of subtle, cumulative, and temporally structured climatic effects that are difficult to reproduce in controlled experiments.

**FIGURE 1:**
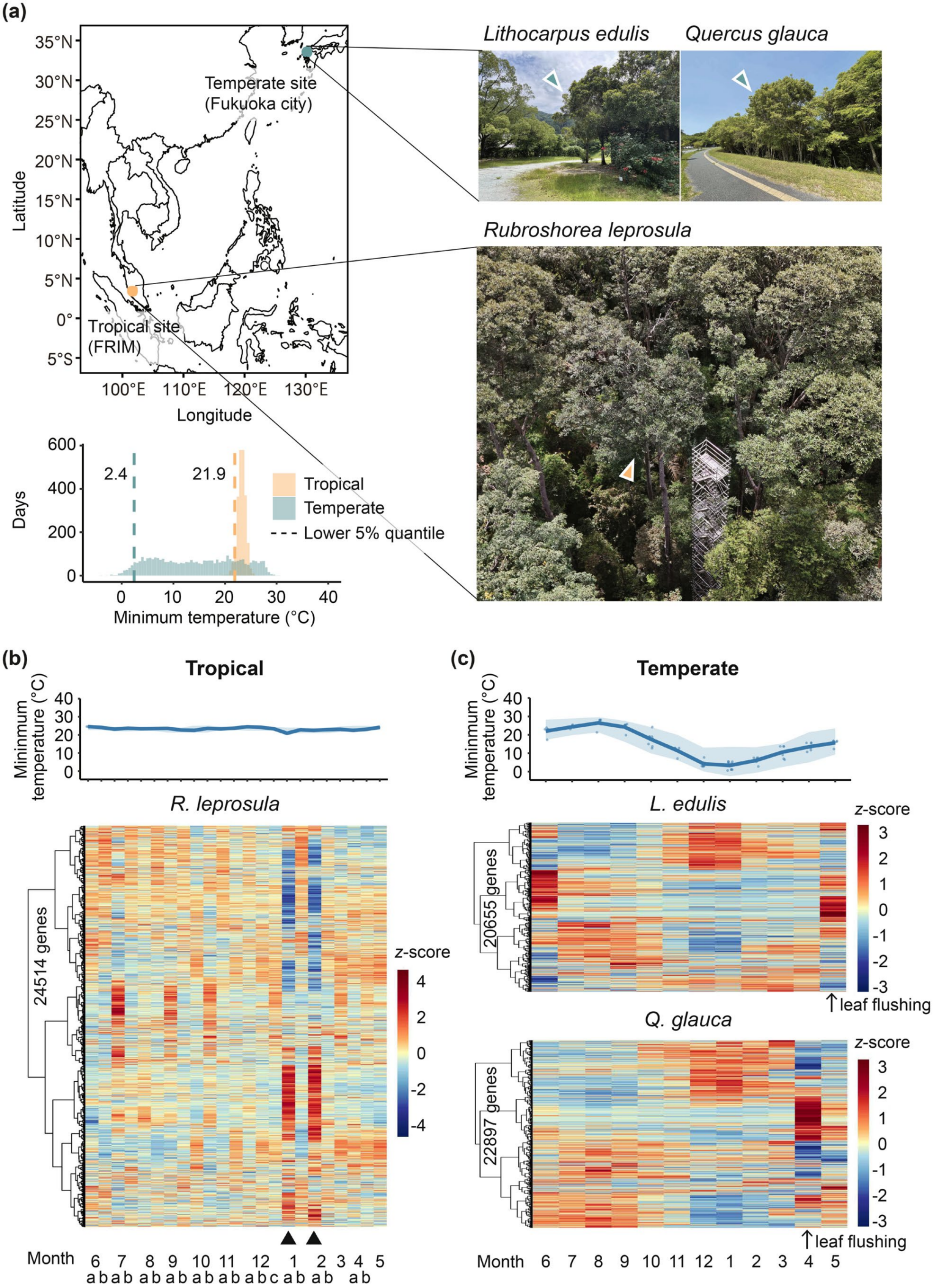
Molecular phenology in tropics and temperate study sites. (a) A map of two study sites, tropical site (FRIM) and temperate site (Fukuoka city). Images of *Lithocarpus edulis*, *Quercus glauca* , and *Rubroshorea leprosula* were presented together with the canopy tower. Each triangle points the species. Arrows in the images indicate the sampled trees. Histograms show the minimum temperatures (2009–2018) at the tropical site (orange) and the temperate site (green), with dashed lines indicating the lower 5% quantile of each distribution. Photo credits: *R. leprosula* (K.K.S. Ng); *L. edulis* and *Q. glauca* (N. Nakata). (b) Seasonal changes in temperature and transcriptional profiles of *R. leprosula*, and (c) *L. edulis* and *Q. glauca* . Different letters (a, b, and c) along the horizontal axis indicate relative sampling order within the same month, progressing from earlier (a) to later (c). Upper panel: Dots show minimum temperatures on each sampling date; lines show monthly averages; shading indicates the monthly dynamic range of minimum temperatures during sampling in tropical and temperate regions. Tropical species: *R. leprosula* (June 13, 2017—May 22, 2018); temperate species: *L. edulis* (October 31, 2019—October 20, 2021) and *Q. glauca* (May 23, 2021–April 21, 2023). Lower panel: The color gradient bar of the heatmap indicates the z‐score of gene expression levels, with red representing high expression and blue representing low expression. Triangles indicate time points with lower minimum temperatures in *R. leprosula*.

To clarify how climatic regimes shape transcriptome dynamics under natural conditions, we performed comparative molecular phenology analysis of tropical tree (*Rubroshorea leprosula*) and temperate trees *Lithocarpus edulis* and *Quercus glauca* (Figure 1a). *R. leprosula* dominates Southeast Asian dipterocarp rainforests, whereas *L. edulis* and *Q. glauca* dominate East Asian temperate forests, giving a clear tropical–temperate contrast. Because all three are long‐lived evergreen canopy trees, differences in transcriptome dynamics can be attributed mainly to climate regime rather than life form. Focusing on orthologous genes shared across species, we analyzed how gene expression dynamics and temperature sensitivity differ between tropical and temperate trees. By doing so, we aim to identify temperature‐responsive functional categories and evaluate whether Janzen's climate variability hypothesis—originally proposed at the level of thermal niches—also holds at the level of gene expression responses.

## 2 Materials and Methods

### 2.1 Study Sites and Sample Collection

*R. leprosula*, a member of the Dipterocarpaceae family, is an evergreen tree species widely distributed across the tropical rainforests of Southeast Asia (Ashton 2003; Symington 1942). Within this family, the genus *Rubroshorea*—formerly classified under *Shorea* (Cvetković et al. 2022)—is among the largest, comprising approximately 100 species. Dipterocarp species are economically important tropical timber trees and have attracted considerable research attention due to their unique reproductive phenology called general flowering—a supra‐annual, community‐wide event often followed by synchronized mass fruiting (Ashton et al. 1988; Sakai et al. 1999; Yasuda et al. 1999; Yeoh et al. 2017; Chen et al. 2018; Numata et al. 2022).

*R. leprosula* samples were collected from Forest Compartment No. 16 at the Forest Research Institute Malaysia (FRIM) (3°14′ N, 101°37′ E, 145 m a.s.l.), located in Selangor, Malaysia (Figure 1a and Table S1). Two individuals were selected for sampling, with diameters at breast height (DBH) of 74 and 65 cm, and an estimated canopy height of approximately 40 m. Leaf samples were collected biweekly from June 2017 to May 2018 using a canopy tower (Figure 1a and Table S1) from each individual from the branches in the emergent canopy layer. During the observation period, both individuals remained in the vegetative state and did not flower. All sampling was conducted around midday. Freshly collected leaves were immediately preserved in tissue storage reagent (RNAlater; Ambion, Austin, TX, USA) and stored at −80°C until further analysis. To examine the relationship between gene expression and abiotic environmental conditions, we used daily maximum, mean, and minimum temperatures, and precipitation monitored at the FRIM KEPONG weather station (3°14′ N, 101°42′ E)—previously reported by Numata et al. (2022). In addition, we incorporated daily solar radiation data measured above the forest canopy at a height of 52 m on an observation tower located approximately 2 km from the 50‐ha plot in the Pasoh Forest Reserve in Malaysia (2°58′ N, 102°18′ E) (Kosugi et al. 2012).

Although comparisons among closely related species would be ideal, forest‐dominant taxa that span broad latitudinal ranges from the tropics to the temperate zone are extremely limited. Because Dipterocarpaceae are absent from temperate regions, we instead selected evergreen Fagaceae species that dominate temperate forests for comparison. RNA‐seq data for *L. edulis* and *Q. glauca* , sampled in Fukuoka, Japan (Figure 1a), were obtained from the previous study (Kudo et al. 2025). These two evergreen tree species are widely distributed across temperate regions of East and Southeast Asia (Govaerts and Frodin 1998). Leaf samples were collected monthly around midday from three *Q. glauca* individuals and one *L. edulis* individual over two years (October 2019 to October 2021 for the *L. edulis* and April 2021 to April 2023 for *Q. glauca* ). Daily maximum, mean, and minimum temperatures were obtained from the Japan Meteorological Agency for Fukuoka City and used to evaluate environmental conditions. For both tropical and temperate sites, the lower 5th percentile values of minimum and maximum temperatures were calculated over a 10‐year period (2009–2018).

### 2.2 RNA Extraction and RNA‐Seq Analysis

Total RNA was extracted from leaf samples collected from *R. leprosula* using hexadecyltrimethylammonium bromide (CTAB) buffer (2% CTAB, 1.4 m NaCl, 20 mm EDTA, 100 mm Tris–HCl pH 8.0, 1% β‐mercaptoethanol) following the protocol described previously (Yeoh et al. 2017). RNA integrity was assessed using the Agilent RNA 6000 Nano kit on a 2100 Bioanalyzer (Agilent Technologies). For each sample, 2.6–3.0 μg of total RNA was sent to Apical Scientific Sdn. Bhd., where cDNA libraries were prepared using a library preparation kit (Eukaryotes Poly‐A tailed) for Illumina. Transcriptome sequencing (150 bp paired‐end reads) was performed on an Illumina NovaSeq 6000 platform (Illumina, San Diego, CA, USA).

Gene expression quantification followed a three‐step pipeline: (1) quality filtering was performed using fastp v0.23.2 with default settings (https://github.com/OpenGene/fastp); (2) RNA‐seq reads were mapped to the reference genome of *R. leprosula* (Satake, Imai, et al. 2024) using STAR 2.7.10b (https://github.com/alexdobin/STAR), with a mean mapping rate of 85.9% ± 2.2% (*n* = 46); and (3) transcript abundance was quantified and the read per kilobase (RPK) value was calculated using RSEM v1.3.1 (https://deweylab.github.io/RSEM/). After filtering, we obtained 9.62 ± 2.46 million paired reads per sample. The overall similarity between two individuals was high, exhibiting a mean correlation coefficient of 0.929 ± 0.0159, calculated from 40,654 *R. leprosula* genes across 46 leaf samples. Genes with a mean RPK ≥ 5 across all samples were considered to be expressed. Out of a total of 40,654 genes, 24,514 genes (60.3%) met this criteria. Next, RPK values were further normalized to GeTMM using edgeR v3.38.4, with normalization factors calculated by calcNormFactors and GeTMM values obtained by cpm. *Z*‐score normalization was conducted by first calculating the mean GeTMM expression level of each gene across the two individuals, transforming them to log2[GeTMM + 1.0], and then standardized to have a mean of zero and a standard deviation of one. Medians and 95% confidence intervals of dynamic range of gene expression were calculated using 1000 bootstrap resamples with the boot package in R (v.4.4.2), applying the percentile method (boot.ci, type = “perc”).

Similar to the analysis of *R. leprosula*, RNA‐seq data from 27 leaf samples of *L. edulis* and 57 leaf samples of *Q. glauca* were filtered using the same criterion (mean RPK ≥ 5 across all samples). The RPK values were then converted to GeTMM using edgeR v4.4.2. In total, 20,655 of 34,059 genes (60.6%) were expressed in *L. edulis* , whereas 22,897 of 39,758 genes (57.6%) were expressed in *Q. glauca* .

### 2.3 Identification of Orthologs

To enable cross‐species comparisons of gene expression, we first determined orthogroups and their corresponding genes using OrthoFinder2 (version 2.5.5) with default settings. We then identified 4682 single‐copy orthologous genes among the three species. From these orthogroups, we extracted 3793 single‐copy orthologous genes that were expressed in all three species (Table S2). These single‐copy orthologs were then used for comparative expression analyses.

### 2.4 Hierarchical Clustering

Hierarchical clustering was performed using expressed genes to assess similarities in gene expression profiles across samples. Log2‐transformed GeTMM values (log2[GeTMM +1]) were averaged across individuals per species and normalized within each species for hierarchical clustering. All expressed genes in each species (*R. leprosula*: 24,514; *L. edulis* : 20,655; *Q. glauca* : 22897) were clustered independently using the Ward's method and Euclidean distance, implemented via the pheatmap package (v. 1.0.12) in R (v. 4.2.0 and 4.4.2), with the options clustering_distance_rows = “euclidean” and clustering_method = “ward.D2”. To compare overall expression patterns among the three species, we used *z*‐score–normalized transcriptome data of the 3793 single‐copy orthologs within each species and performed hierarchical clustering across both samples and genes.

### 2.5 Relationship Between Abiotic Factors and Transcriptional Profile

We examined the relationship between gene expression and temperature using linear models implemented in the lm function in R (version 4.4.2). Log2[GeTMM + 1.0]‐transformed gene expression levels were used as the response variable, and minimum temperature was used as the explanatory variable. Genes showing significant positive or negative relationships with minimum temperature were identified based on adjusted *p‐*values using the Benjamini–Hochberg method (FDR < 0.05; Benjamini and Hochberg 1995).

To assess the relative sensitivity to temperature fluctuations between the tropical and temperate species, we examined the relationships between gene expression and minimum temperature in tropical and temperate tree species using linear models. For each gene, log2[GeTMM + 1]‐transformed expression levels were used as the response variable, while minimum temperature, species, and their interaction were included as explanatory variables. We also constructed an additional set of linear models in which the minimum temperature was standardized prior to analysis. Specifically, minimum temperatures from both tropical and temperate sites, covering the 10‐year period preceding the last sampling date for each species, were transformed to have a mean of zero and a standard deviation of one, allowing direct comparison of temperature sensitivity across species independent of absolute temperature range. Genes that showed significant differences in either the main effect of minimum temperature or the interaction between minimum temperature and species between the two types of models were extracted and defined as temperature‐responsive genes.

We calculated the ratios of regression coefficients for *R. leprosula* (*β*_R_) relative to those of *L. edulis* (*β*_L_) and *Q. glauca* (*β*_Q_). Each ratio was computed using the absolute values of the corresponding regression coefficients to compare the sensitivity of gene expression to temperature among species (*β*_R_/*β*_L_, *β*_R_/*β*_Q_, *β*_Q_/*β*_L_, and *β*_L_/*β*_Q_). Differences in these ratios were evaluated using paired *t*‐tests. We calculated these ratios for both sets of linear models, in which either the raw minimum temperature or the normalized minimum temperature was used as the explanatory variable.

### 2.6 Gene Ontology (GO) Enrichment Analysis

To perform GO enrichment analysis for each temperature responsive gene, we first annotated GO terms for each gene using BLASTX searches (BLAST version 2.6.0+) of the *R. leprosula* protein database (Satake, Hagiwara, et al. 2024; Satake, Imai, et al. 2024) against the *Arabidopsis thaliana* protein dataset (Araport11_201606) with an E‐value cutoff of < 1e‐10. The top hit for each query was used for annotation using the *A. thaliana* GO database from Dicots PLAZA 5.0 (Release 2021–09). The same procedure was applied to *L. edulis* and *Q. glauca* .

GO term enrichment analysis was conducted using a conditional hypergeometric test implemented via the hyperGTest function in the GOstats package (version 2.72.0; Falcon and Gentleman 2007) in R (version 4.4.2). The analysis was performed with the following parameters: ontology = “BP”, pvalueCutoff = 0.05, conditional = TRUE, and testDirection = “over”. The background gene set (gene universe) for the enrichment analysis comprised 24,514 *R. leprosula* genes, 20,655 *L. edulis* genes, and 22,897 *Q. glauca* genes. *p*‐values for each GO term were adjusted using the Benjamini–Hochberg method (Benjamini and Hochberg 1995) via the *p*.adjust function with the option “BH” in R (version 4.4.2). GO terms with adjusted *p‐*values < 0.05 were considered statistically significant. Among these, the top 5 terms with the lowest adjusted *p*‐values were selected for further representation.

### 2.7 Calculation of Dynamic Range

To evaluate the relationship between temperature variability and gene expression variability, we calculated the annual dynamic range of temperature and that of gene expression for both tropical and temperate sites. The dynamic range of log2‐transformed gene expression levels was defined as the difference between the maximum and minimum values observed during the sampling period.

## 3 Results

### 3.1 Comparison of Transcriptome Profiles

Comparison of genome‐wide gene expression profiles across a full annual cycle between the tropical (*R. leprosula*) and the temperate species ( *L. edulis* and *Q. glauca* ) revealed distinct differences. In the tropical species, expression changes were sporadic and lacked clear periodicity (Figure 1b), whereas both the temperate species exhibited pronounced annual cycles, with a clear distinction between winter and other seasons reported previously (Figure 1c; Kudo et al. 2025). The sporadic transcriptional pattern in the tropical species was consistent across both sampled individuals (Figure S1), indicating that the observed pattern was robust and reproducible.

The most pronounced changes in gene expression in the tropical species occurred on January 11 and February 12 (triangles in Figure 1b). On these two dates, the daily minimum temperatures were 20.9°C and 22.5°C, respectively (Figure S2 and Table S1)—values that fall near the lower 5% quantile (21.9°C) of the 10‐year distribution of daily minima at the Kepong meteorological station in Malaysia (Figure 1a)—indicating that both sampling events coincided with unusually low temperatures for this site. Indeed, the minimum temperatures on these two sampling dates were significantly lower than the lower 5% quantile derived from 10,000 random resamplings of two sampling dates, whereas maximum temperature, precipitation, and solar radiation fell within the 95% confidence interval of the null distribution (Figure S3).

### 3.2 Seasonal Transcriptional Modes Revealed by Cross‐Species Clustering

**FIGURE 2:**
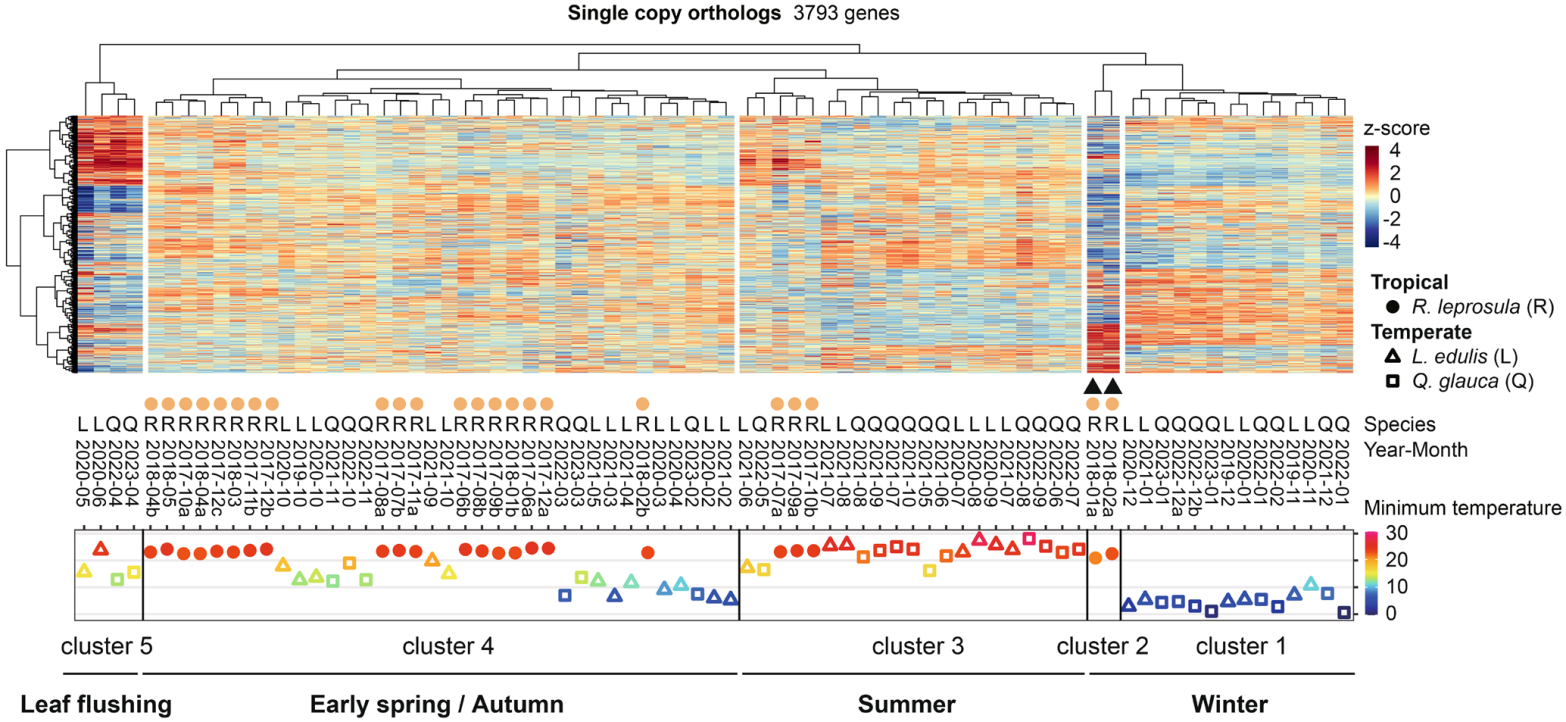
Characterization of seasonal gene expression patterns in tropical and temperate species. Hierarchical clustering of seasonal expression profiles for 3793 single copy orthologous genes across three species: *Rubroshorea leprosula* (R), *Lithocarpus edulis* (L), and *Quercus glauca* (Q). The minimum temperature on each sampling date is also plotted for reference. Triangles indicate time points with lower minimum temperatures in *R. leprosula*.

Among the 24,514 genes identified in *R. leprosula*, 3793 corresponded to single‐copy orthologs shared with the two temperate species, *L. edulis* and *Q. glauca* . To compare overall expression patterns among the three species, we normalized the transcriptome data of the 3793 single‐copy orthologs within each species, combined the datasets, and performed hierarchical clustering across samples and genes. This hierarchical clustering across samples identified five distinct transcriptional modes (Figure 2). Cluster 1 comprised samples collected from temperate winter (Nov–Feb), while cluster 2 contained tropical samples exhibiting marked transcriptional shifts in response to a slight temperature decrease (indicated as solid triangles). Notably, the low‐temperature response observed in *R. leprosula* (cluster 2) clustered closely with the winter transcriptional mode in the temperate site (cluster 1). These results suggest that the response to temperature drops around 21°C in the tropical species parallels the winter transcriptional profile of the temperate site.

Cluster 3 consisted primarily of temperate summer (May–Oct) with partial overlap from tropical samples. Cluster 4 represented a mixture of temperate spring and autumn samples (Feb–May/Sep–Nov) together with the majority of tropical samples, whereas cluster 5 included temperate samples corresponding to the period of leaf flushing at the temperate site (Kudo et al. 2025). Because the majority of *R. leprosula* profiles most closely matched the temperate spring–autumn modes (cluster 4 in Figure 2), the overall transcriptional states in the tropical species resembled those of spring and autumn in the temperate site.

### 3.3 Characterization of Genes Associated With Temperature Responses

**FIGURE 3:**
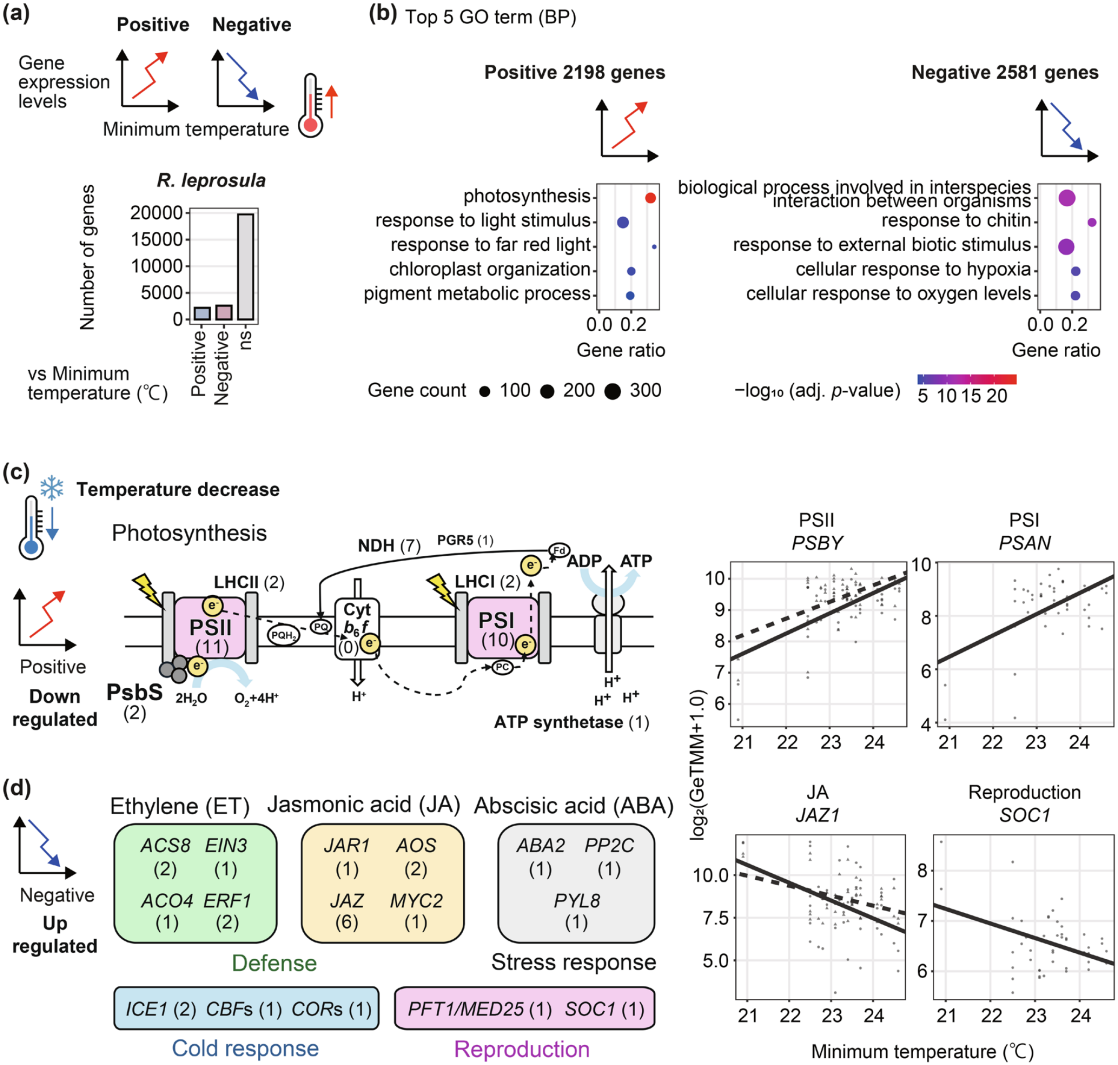
Transcriptional responses of *Rubroshorea leprosula* to changes in minimum temperature. (a) Number of *R. leprosula* genes showing transcriptional responses to minimum temperature. The response type is classified as “Positive” (expression increases with higher temperature), “Negative” (expression decreases with higher temperature), or “ns” (no significant change). (b) The Top 5 GO terms for biological processes for genes significantly responsive to minimum temperature in *R. leprosula*. The size of each dot reflects the gene count, and the color scale represents the adjusted *p*‐value for enrichment, with brighter colors indicating higher statistical significance. Gene ratio was calculated as the number of significantly responsive genes associated with each GO term divided by the total number of genes annotated to that term. (c) Candidate photosynthetic pathways in the tropical tree showing positive correlations with temperature and (d) stress‐response pathways showing negative correlations. For each pathway, the number of corresponding genes in *R. leprosula* is given in parentheses, along with the gene name. The detailed gene lists are provided in Table S5. Results of the linear regression for representative genes are also shown. Regression lines were drawn using estimated coefficients from the linear model. Representative genes include a small *photosystem II subunit* (*PSBY*), *photosystem I reaction center subunit N* (*PSAN*), *jasmonate ZIM‐domain protein 1* (*JAZ1*), and *SOC1*, also known as *AGAMOUS‐LIKE 20* (*AGL20*).

To quantify the temperature sensitivity of gene expression and to characterize genes exhibiting sporadic changes associated with drops in minimum temperature in the tropical species, we fitted linear models of expression of 24,514 *R. leprosula* genes against temperature. Of these, 2198 genes showed positive associations with temperature, whereas 2581 genes showed negative associations (Figure 3a and Table S3). GO enrichment analysis indicated that the positively associated genes were enriched for photosynthesis‐related terms (Figure 3b and Table S4), suggesting that photosynthetic processes tend to be suppressed when temperature dropped below approximately 22°C. By contrast, negatively associated genes were enriched for stress‐response GO terms (Figure 3b and Table S4).

From the genes that exhibited decreased expression to slight decreases in temperature, filtering by photosynthesis‐related GO annotations identified a broad set of photosynthesis‐related genes, including those encoding photosystem I (PSI) and II (PSII), light‐harvesting antenna complexes (LHCI and LHCII), and components involved in photoprotection and electron transport, such as photosystem II subunit S (PsbS), NDH complex subunits, PGR5, and ATP synthase (Figure 3c and Table S5). This finding suggests a coordinated, system‐wide regulation of the photosynthetic machinery—from light capture to electron transport and photophosphorylation—in response to a subtle drop in temperature. Conversely, among the genes up‐regulated by a small temperature decrease, we detected not only components of ethylene (ET), jasmonate (JA), and abscisic acid (ABA) biosynthesis and signaling, but also cold‐response genes and flowering‐promoting factors (Figure 3c and Table S5; *MEDIATOR subunit 25* [*MED25*/*PFT1*] and *SUPPRESSOR OF OVEREXPRESSION OF CONSTANS 1* [*SOC1*]). These patterns suggest that even mild decreases in temperature elicit an integrated reconfiguration of the cold‐response and multi‐hormone (ET–JA–ABA) networks, together with pathways associated with reproduction.

We compared temperature‐responsive gene expression between the tropical and temperate species. In the temperate trees, more genes showed significant associations with temperature ( *L. edulis* : 3607 positive and 3619 negative, *Q. glauca* : 5156 positive and 5276 negative; Figure S4a and Table S3). Among the temperature‐responsive genes identified in the tropical species, 21.9% overlapped with those in *L. edulis* and 24.4% with those in *Q. glauca* . Across all three species, among 3793 single‐copy orthologs, only 222 genes (5.6%) were shared as temperature‐responsive (73 consistently positive and 50 consistently negative), indicating that the conservation of temperature sensitivity is limited. GO enrichment analysis showed that positively associated genes in the tropical species were enriched for photosynthesis‐related GO terms (Figure S4b and Table S4), as in the tropical species, whereas negatively associated genes in both temperate trees were enriched for metabolic processes rather than stress‐response, in contrast to the tropical species (Figure S4b). Together, these results indicate that mild temperature variation elicits a largely species‐specific transcriptional program, with a conserved photosynthetic up‐regulation but a temperate‐specific shift toward broad metabolic reprogramming rather than stress signaling in the temperate species.

### 3.4 Temperature Sensitivity of Gene Expression Differs Between Tropical and Temperate Species

**FIGURE 4:**
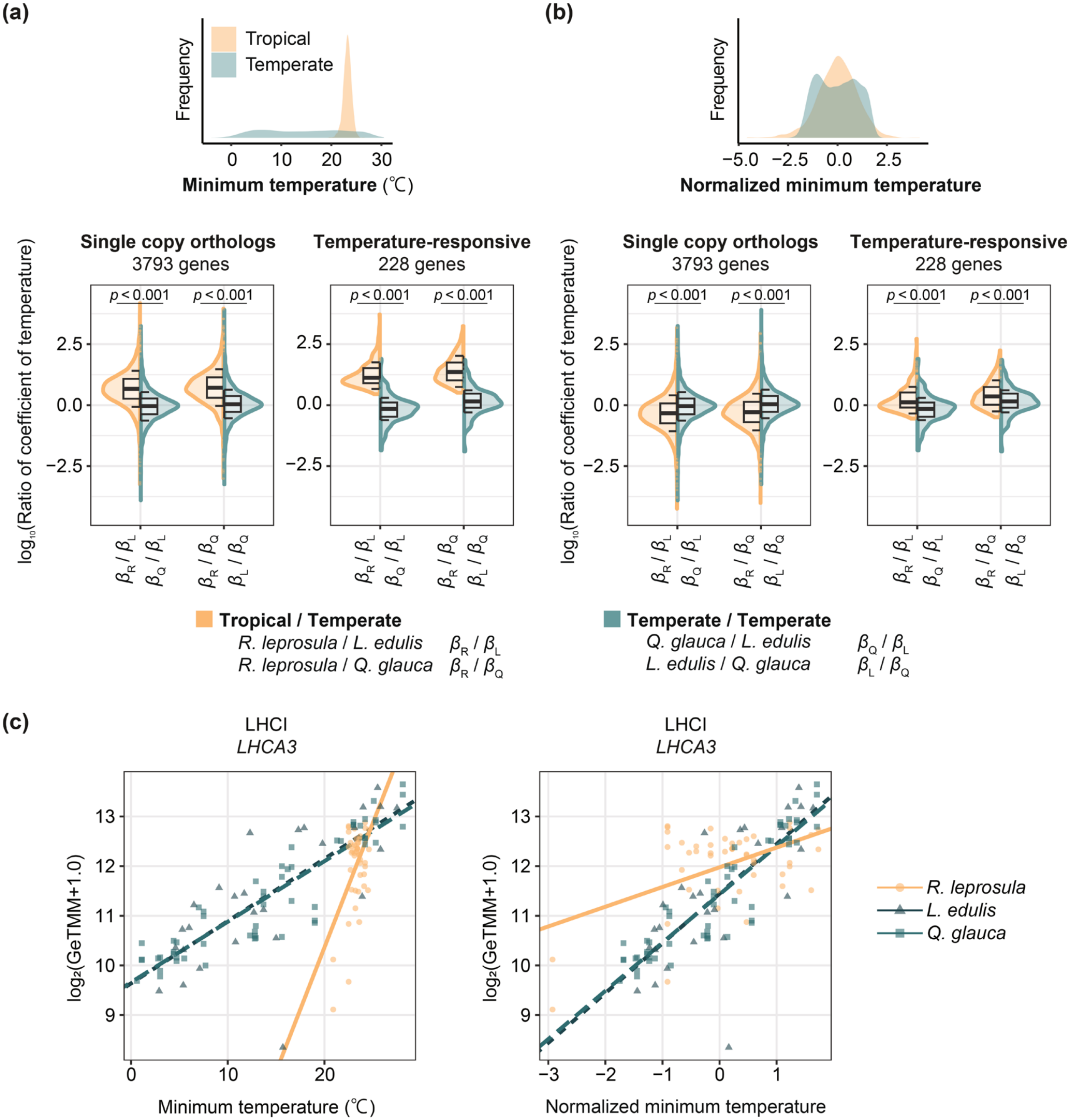
Distribution of the regression coefficient ratios. Violin plots of log10‐transformed absolute ratios from models using (a) minimum temperature and (b) normalized minimum temperature as explanatory variables. *P*‐values from paired *t*‐test are shown. Density plots above illustrate the distributions of temperatures at the tropical (orange) and temperate (green) sites. (c) Linear regression results between gene expression and temperature are shown for minimum and normalized minimum temperatures. As an example, a representative photosynthesis‐related gene, *PSI type III chlorophyll a/b‐binding protein* (*LHCA3*), annotated to photosynthetic pathways, was selected from the 228 temperature‐responsive genes.

To assess cross‐species sensitivity to temperature fluctuations, we took the absolute and normalized values of the regression coefficients of linear models of gene expression against minimum temperature for each species (Figure 4a,b). Using absolute regression coefficients, 577 genes showed significant associations, whereas using normalized minimum temperature, 239 genes showed significant associations (Table S6). Among these, 228 genes were common to both analyses and were defined as temperature‐responsive genes (Table S7).

Based on linear models using minimum temperature as the explanatory variable, the median ratios of regression coefficients for the 3793 single‐copy orthologous genes between the tropical and temperate species were 5.18 and 4.67 times greater than those observed between the temperate species pairs (Figure 4a, *β*_R_/*β*_L_ vs. *β*_Q_/*β*_L_: paired *t*‐test: *p* < 0.001, *β*_R_/*β*_Q_ vs. *β*_L_/*β*_Q_: paired *t*‐test: *p* < 0.001). When the analysis was restricted to the 228 temperature‐responsive genes in all species, the median ratios between the tropical and temperate species were 22.9 and 13.2 times greater than those observed between the temperate species pairs (Figure 4a, *β*_R_/*β*_L_ vs. *β*_Q_/*β*_L_: paired *t*‐test: *p* < 0.001, *β*_R_/*β*_Q_ vs. *β*_L_/*β*_Q_: paired *t*‐test: *p* < 0.001).

When we normalized temperature within each site (mean = 0, SD = 1) and refit the linear models using the normalized values, the apparent sensitivity gap between the tropical and temperate trees decreased to only 0.517 and 0.465 for the 3793 single‐copy orthologous genes (Figure 4b, *β*_R_/*β*_L_ vs. *β*_Q_/*β*_L_: paired *t*‐test: *p* < 0.001, *β*_R_/*β*_Q_ vs. *β*_L_/*β*_Q_: paired *t*‐test: *p* < 0.001) and 2.29 and 1.32 for the 228 temperature‐responsive genes (Figure 4b, *β*_R_/*β*_L_ vs. *β*_Q_/*β*_L_: paired *t*‐test: *p* < 0.001, *β*_R_/*β*_Q_ vs. *β*_L_/*β*_Q_: paired *t*‐test: *p* < 0.001). As an example, a representative photosynthesis‐related gene, PSI type III chlorophyll a/b‐binding protein (*LHCA3*), annotated to photosynthetic pathways (Figure 4c), exhibited a 10.1‐fold decrease in *β*_R_/*β*_L_ (from 3.99 to 0.397) when using normalized instead of absolute temperatures, whereas *β*_Q_/*β*_L_ remains nearly unchanged (from 0.973 to 0.970). This suggests that transcriptomic responses scale with relative temperature anomalies from the local baseline rather than with absolute temperature per se.

### 3.5 Comparison of Dynamic Range in Gene Expression Between Tropical and Temperate Species

**FIGURE 5:**
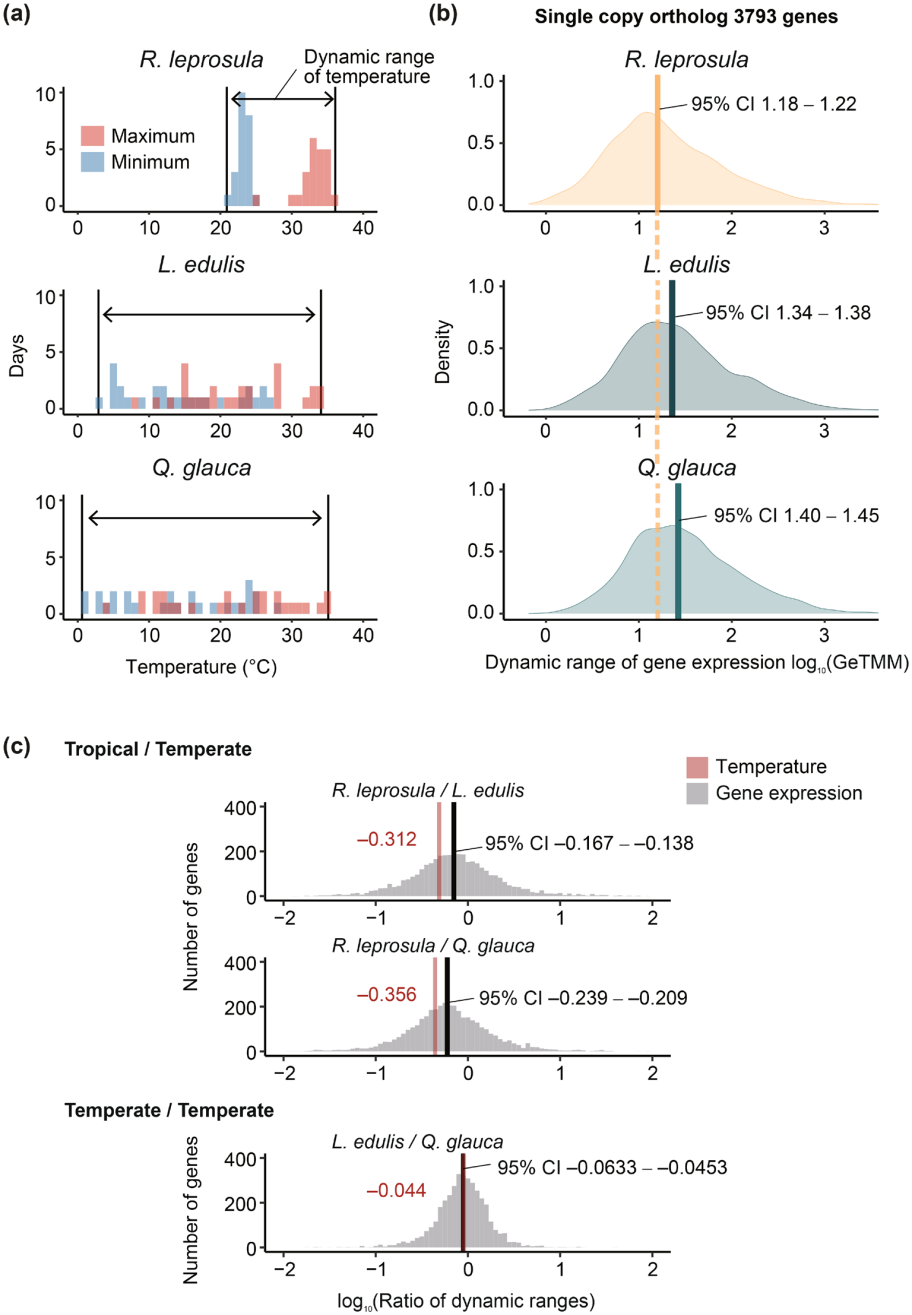
Dynamic range of temperature and gene expression. (a) Temperature distribution during sampling periods for *Rubroshorea leprosula*, *Lithocarpus edulis*, and *Quercus glauca* . Red histograms represent daily maximum temperature, and blue histograms represent daily minimum temperature on the actual sampling dates. Black vertical lines indicate the maximum and minimum temperatures recorded on the sampling days, with arrows showing their dynamic range of temperature. (b) Distribution of dynamic range of 3793 single‐copy gene expression in *R. leprosula*, *L. edulis* , and *Q. glauca*. Each dot represents the expression level of a gene in individual samples and the black dot indicates the mean of expression levels for each gene. The values represent the 95% confidence intervals (CI) of the median log_10_‐transformed dynamic ranges of gene expression. The orange dashed line indicates the 95% CI of the dynamic range of gene expression in *R. leprosula*. (c) Dynamic range ratio of temperature and gene expression across three species. Values in red represent the log_10_‐transformed ratios of dynamic ranges of temperature, while values in black represent the 95% CI of the median log_10_‐transformed ratios of dynamic ranges of gene expression.

To examine the relationship between temperature variability and gene expression variability between the tropical and temperate study sites, we calculated the dynamic range of temperature (Figure 5a) and compared it with the dynamic range of expression levels for the 3793 single‐copy orthologs (Figure 5b). The annual dynamic range of temperature in the tropical site was about half that of the temperate study site (Figure 5a and Table 1), whereas the median dynamic range of gene expression in the tropical species was only 0.84–0.88 times smaller than those of the temperate species (Figure 5b).

**TABLE 1:** Temperature range on the sampling date and sampling time.

Species	Range temperature (max–min) on the sampling date	Annual range temperature (max–min) at sampling period	Sampling period
*Rubroshorea leprosula*	15.2 (36.1–20.9)	16.3 (37.2 to 20.9)	332 days (June 13, 2017—May 22, 2018)
*Lithocarpus edulis*	31.2 (34.1–2.9)	40.2 (38.0 to −2.2)	721 days (October 31, 2019—October 20, 2021)
*Quercus glauca*	34.5 (35.1–0.6)	39.4 (36.9 to −2.5)	699 days (May 23, 2021—April 21, 2023)

To further compare the dynamic ranges between tropical and temperate species, we examined the ratios of gene expression dynamic ranges of tropical species relative to those of temperate species, as well as the corresponding ratios of temperature dynamic ranges (Figure 5c). The 95% confidence intervals of the median log10‐transformed interspecific ratios of dynamic range and the corresponding log10‐transformed ratios for minimum temperature showed that, in temperate species, the dynamic range of gene expression did not exceed the magnitude of temperature variations (Figure 5c). By contrast, in tropical species, the dynamic range of gene expression was significantly greater than that of temperature between the two sites (Figure 5c). These findings suggest that transcriptional changes per unit of temperature variation are amplified and more variable in tropical species.

## 4 Discussion

Our comparative field transcriptomic analysis provides new evidence that tropical trees exhibit high sensitivity to temperature fluctuations and limited capacity to buffer their physiological functions against temperature fluctuations, in contrast to temperate trees that display seasonally coordinated gene expression patterns. The most striking finding was that modest declines in minimum temperature (down to 21°C–22°C) triggered widespread transcriptional changes in the tropical species *R. leprosula*, particularly the down‐regulation of photosynthesis‐related genes and the up‐regulation of stress‐response genes (Figure 3). This sensitivity is remarkable given that such temperatures are well within the normal seasonal range of temperate species, yet they represent rare, extreme events in the tropics. These results extend Janzen's climate variability hypothesis (Janzen 1967) to the molecular level, demonstrating that narrow thermal niches in tropical species are mirrored by restricted transcriptional tolerance to environmental fluctuations.

Tropical trees generally exhibit weak acclimation to cold, such that even modest cooling suppresses the photosynthetic machinery and makes it prone to photoinhibition (Huang et al. 2010; Nievola et al. 2017). Consistently, photosynthesis–temperature curves of tropical forest species are skewed toward higher temperatures, with pronounced performance declines at low temperatures and only limited plasticity toward the cold end (Mori et al. 1990; Slot and Winter 2017). By contrast, winter adjustments of photosynthesis and photoprotective mechanisms have been repeatedly documented in Mediterranean *Q. ilex* and Japanese evergreen oaks (Hikosaka et al. 1999; Corcuera et al. 2005; Kosugi and Matsuo 2006; Miyazawa and Kikuzawa 2006; Camarero et al. 2012). These patterns are congruent with our observation of reduced gene expression and physiological plasticity in the weakly seasonal tropics relative to strongly seasonal temperate climates. Because carbon gain is tightly coupled to survival and reproductive output, the inability to maintain photosynthetic function during occasional cold events may directly constrain the realized niche of tropical trees. Although photosynthetic activity was not directly assessed in this study, future work combining physiological measurements with gene expression analyses will be essential to link transcriptional changes to functional consequences.

Our data show that relatively modest drops in minimum temperature coincided with induction of stress‐response pathways and with the up‐regulation of a putative MED25/PFT1 ortholog in *R. leprosula*. Because *MED25/PFT1* has been implicated in flowering‐time control downstream of phyB in *A. thaliana* (Iñigo et al. 2012; Elfving et al. 2011; Yao et al. 2019) and functions as a co‐activator of jasmonate (JA) signaling at COI1–JAZ–MYC hubs (An et al. 2017; Zhai and Li 2019), this result provides a plausible mechanistic link between stress signaling and the floral transition in Dipterocarpaceae. Dipterocarps show general flowering, known as supra‐annual, community‐wide mass flowering and fruiting events (Sakai et al. 1999). General flowering in Southeast Asian dipterocarps is a supra‐annual, climate‐cued phenomenon in which cool spells and drought act—often synergistically—to trigger community‐wide floral induction (Yeoh et al. 2017; Chen et al. 2018; Numata et al. 2022; Satake et al. 2022). Previous reports show that low night‐time temperature events (often < 20°C) typically precede major general flowering by ~1–2 months (Ashton et al. 1988; Yasuda et al. 1999; Numata et al. 2003; Brearley et al. 2007), yet the molecular mechanisms remain unresolved. We propose that modest cooling up‐regulates a *PFT1/MED25* and *SOC1* ortholog in *R. leprosula* thereby feeding temperature signals into flowering circuits. Although flowering was not recorded during the sampling period, extended molecular‐phenology observations in tropical sites should capture flowering episodes and elucidate their regulatory mechanisms in future studies.

While much of the climate change discourse has focused on rising temperatures, our results highlight the importance of considering minimum temperatures and their variability, which can be equally consequential for tropical species. If future climates bring not only warming but also greater temperature variability, tropical trees may face compounding risks: chronic stress from high temperatures and acute stress from anomalous cold events. Because this study examined a single tropical species against two temperate comparators, broader generalization will require expanding both the taxonomic and geographic scope in future work.

## Funding

This study was supported by Japan Society for the Promotion of Science (23H04965, 23H04966).

## Conflicts of Interest

The authors declare no conflicts of interest.

## Supporting information


**Figure S1:** Heatmaps of seasonal gene expression profiles for 24,514 genes of the tropical tree *R. leprosula* (FRIM, Malaysia). Different letters (a, b, and c) on the horizontal axis indicate relative earliness within the same sampling month. The color gradient bar indicates the z‐score of gene expression levels, with red representing high expression and blue representing low expression. Triangles indicate time points with lower minimum temperatures in *R. leprosula*.
**Figure S2:** Maximum (red), mean (black) and minimum (blue) temperature, daily precipitation at the Kepong meteorological station, and daily solar radiation at the Pasoh Forest Reserve in Malaysia. Dotted lines indicate each sampling date. Triangles indicate time points with lower minimum temperatures in *R. leprosula*.
**Figure S3:** Histograms show the distributions of 10,000 randomly sampled pairs of minimum, mean, and maximum temperatures, precipitation, and solar radiation at FRI Kepong. The solid red line indicates the mean of two low‐temperature sampling dates (January a and February a), and the dashed black line shows the lower 5% quantile of the distribution with its value. Distributions were generated by randomly sampling two observations with replacement 10,000 times and calculating their means (permutation test).
**Figure S4:** Transcriptional responses to minimum temperature and GO enrichment analysis in two temperate species. (a) Number of genes showing transcriptional responses to minimum temperature in *L. edulis* and *Q. glauca* . The response type is classified as “Positive” (expression increases with higher temperature), “Negative” (expression decreases with higher temperature), or “ns” (no significant change). (b) GO enrichment analysis of genes significantly responsive to minimum temperature in *L. edulis* and *Q. glauca* , including 3607 *L. edulis* and 5156 *Q. glauca* , genes with increased expression (“Positive”) and 3619 *L. edulis* and 5276 *Q. glauca* , genes with decreased expression (“Negative”). The plot displays the top 5 significantly enriched Gene Ontology (GO) terms related to biological processes. The size of each dot reflects the gene count, and the color scale represents the adjusted *p*‐value for enrichment, with blighter colors indicating higher statistical significance. Gene ratio was calculated as the number of significantly responsive genes associated with each GO term divided by the total number of genes annotated to that term.

**Table S1:** Sample information for the tropical tree species *R. leprosula*.
**Table S2:** Orthogroup list of expressed genes of *R. leprosula*, *L. edulis* , and *Q. glauca* used in Figure 2.
**Table S3:** List of temperature‐responsive genes of *R. leprosula*, *L. edulis* , and *Q. glauca* shown in Figure 3a and Figure S4a, respectively.
**Table S4:** Top 30 GO terms for temperature‐responsive genes of *R. leprosula*, *L. edulis* , and *Q. glauca* shown in Figure 3b and Figure S4b, respectively.
**Table S5:** Gene list of genes related to photosynthesis and stress responses presented in Figure 3c.
**Table S6:** Temperature‐responsive genes (577 absolute, 239 normalized temperature) among the 3793 single‐copy orthologous genes used to compare temperature sensitivity in response to minimum temperature between tropical and temperate species in Figure 4.
**Table S7:** List of 228 temperature‐responsive genes identified across tropical and temperate species shown in Figure 4.

## Data Availability

The sequence data supporting the findings of this study have been deposited in the DNA Data Bank of Japan (DDBJ) Sequence Read Archive (DRA) database under BioProject accession number PRJDB37847, with BioSample accession numbers SAMD01695826–SAMD01695871.
