# Supplementary material for: A Cross‐Climate Comparison of Molecular Phenology in Three Tropical and Temperate Trees
Source: Plant Environ Interact. 2026 Apr 9;7(2):e70146. doi: 10.1002/pei3.70146 (PMC13063121; doi:10.1002/pei3.70146)
Supplement: Supplementary file 1 — Figure S1: Heatmaps of seasonal gene expression profiles for 24,514 genes of the tropical tree R. leprosula (FRIM, Malaysia). Different letters (a, b, and c) on the horizontal axis indicate relative earliness within the same sampling month. The color gradient bar indicates the z‐score of gene expression levels, with red representing high expression and blue representing low expression. Triangles indicate time points with lower minimum temperatures in R. leprosula. Figure S2: Maximum (red), mean (black) and minimum (blue) temperature, daily precipitation at the Kepong meteorological station, and daily solar radiation at the Pasoh Forest Reserve in Malaysia. Dotted lines indicate each sampling date. Triangles indicate time points with lower minimum temperatures in R. leprosula. Figure S3: Histograms show the distributions of 10,000 randomly sampled pairs of minimum, mean, and maximum temperatures, precipitation, and solar radiation at FRI Kepong. The solid red line indicates the mean of two low‐temperature sampling dates (January a and February a), and the dashed black line shows the lower 5% quantile of the distribution with its value. Distributions were generated by randomly sampling two observations with replacement 10,000 times and calculating their means (permutation test). Figure S4: Transcriptional responses to minimum temperature and GO enrichment analysis in two temperate species. (a) Number of genes showing transcriptional responses to minimum temperature in L. edulis and Q. glauca . The response type is classified as “Positive” (expression increases with higher temperature), “Negative” (expression decreases with higher temperature), or “ns” (no significant change). (b) GO enrichment analysis of genes significantly responsive to minimum temperature in L. edulis and Q. glauca , including 3607 L. edulis and 5156 Q. glauca , genes with increased expression (“Positive”) and 3619 L. edulis and 5276 Q. glauca , genes with decreased expression (“Negative”). The plo [file PEI3-7-e70146-s002.docx]

Supplementary

**Title:　 A cross-climate comparison of molecular phenology in three tropical and temperate trees**

Authors: Atsuko Miyawaki-Kuwakado^1^, Nakata Taichi^1^, Yuka Ikezaki^1^, Naoki Tani^2,3^, Yoshiko Kosugi^4^, Kevin Kit Siong Ng^5^, Soon Leong Lee^5^, Akiko Satake^1*^

Affiliations:

^1^ Department of Biology, Faculty of Science, Kyushu University, Fukuoka, Japan

^2^ Forestry Division, Japan International Research Center for Agricultural Sciences, Tsukuba, Ibaraki, Japan

^3^ Institute of Life and Environmental Sciences, University of Tsukuba, Tsukuba, Ibaraki, Japan

^4^ Graduate School of Agriculture, Kyoto University, Kyoto 606-8502, Japan

^5^ Forestry Biotechnology Division, Forest Research Institute Malaysia, Selangor, Malaysia


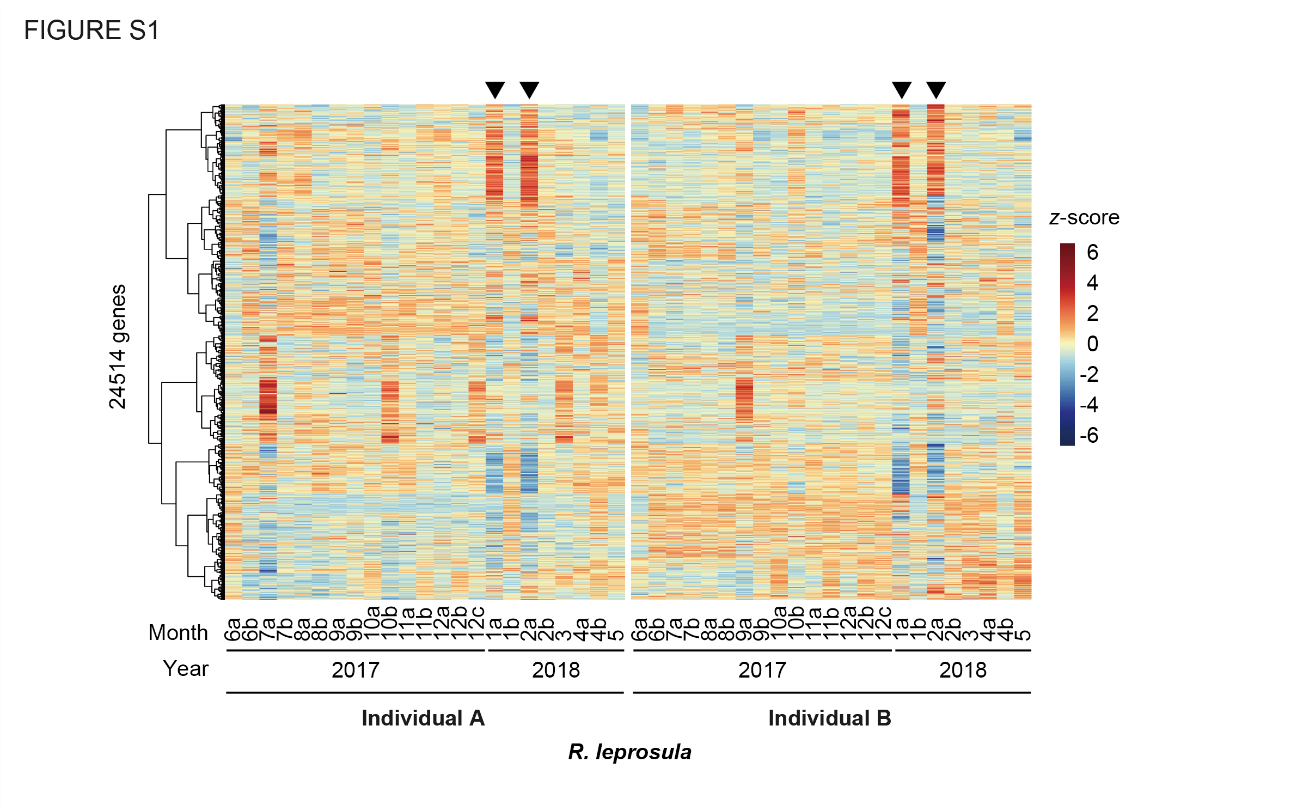


**FIGURE S1** Heatmaps of seasonal gene expression profiles for 24514 genes of the tropical tree *R. leprosula* (FRIM, Malaysia). Different letters (a, b, and c) on the horizontal axis indicate relative earliness within the same sampling month. The colour gradient bar indicates the z-score of gene expression levels, with red representing high expression and blue representing low expression. Triangles indicate time points with lower minimum temperatures in *R. leprosula*.


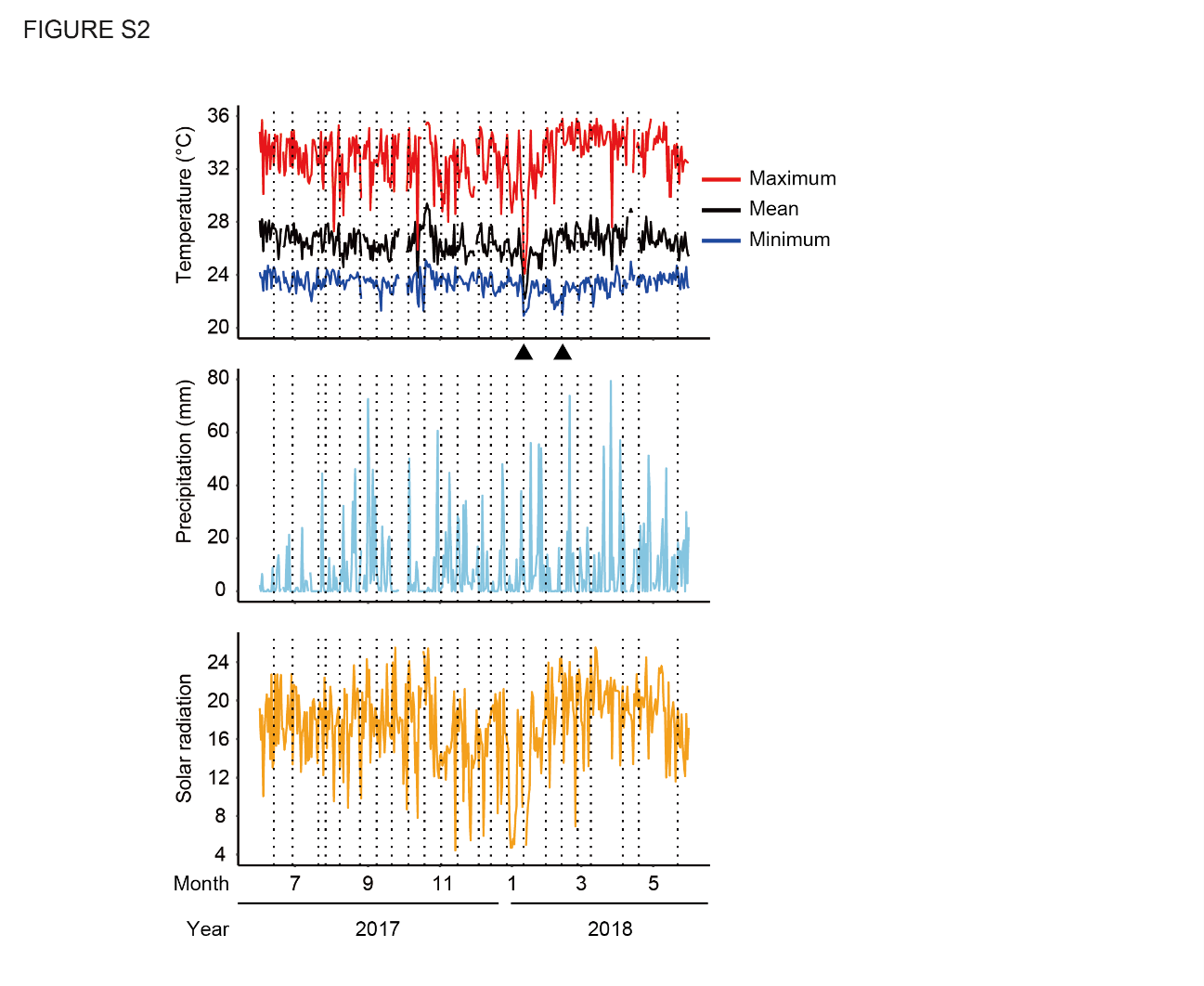


**FIGURE S2** Maximum (red), mean (black) and minimum (blue) temperature, daily precipitation at the Kepong meteorological station, and daily solar radiation at the Pasoh Forest Reserve in Malaysia. Dotted lines indicate each sampling date. Triangles indicate time points with lower minimum temperatures in *R. leprosula*.


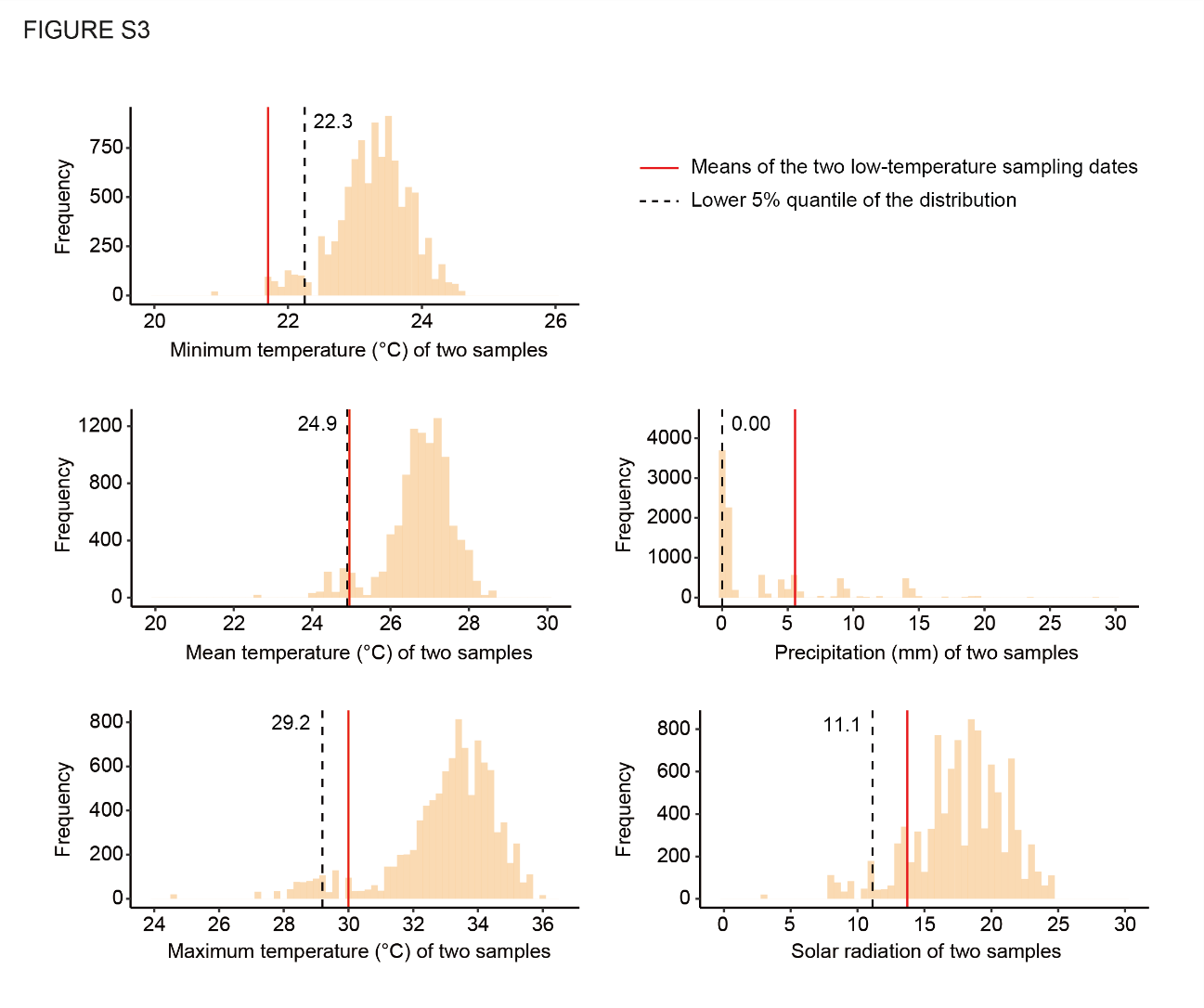


**FIGURE S3** Histograms show the distributions of 10000 randomly sampled pairs of minimum, mean, and maximum temperatures, precipitation, and solar radiation at FRI Kepong. The solid red line indicates the mean of two low-temperature sampling dates (January a and February a), and the dashed black line shows the lower 5% quantile of the distribution with its value. Distributions were generated by randomly sampling two observations with replacement 10000 times and calculating their means (permutation test).


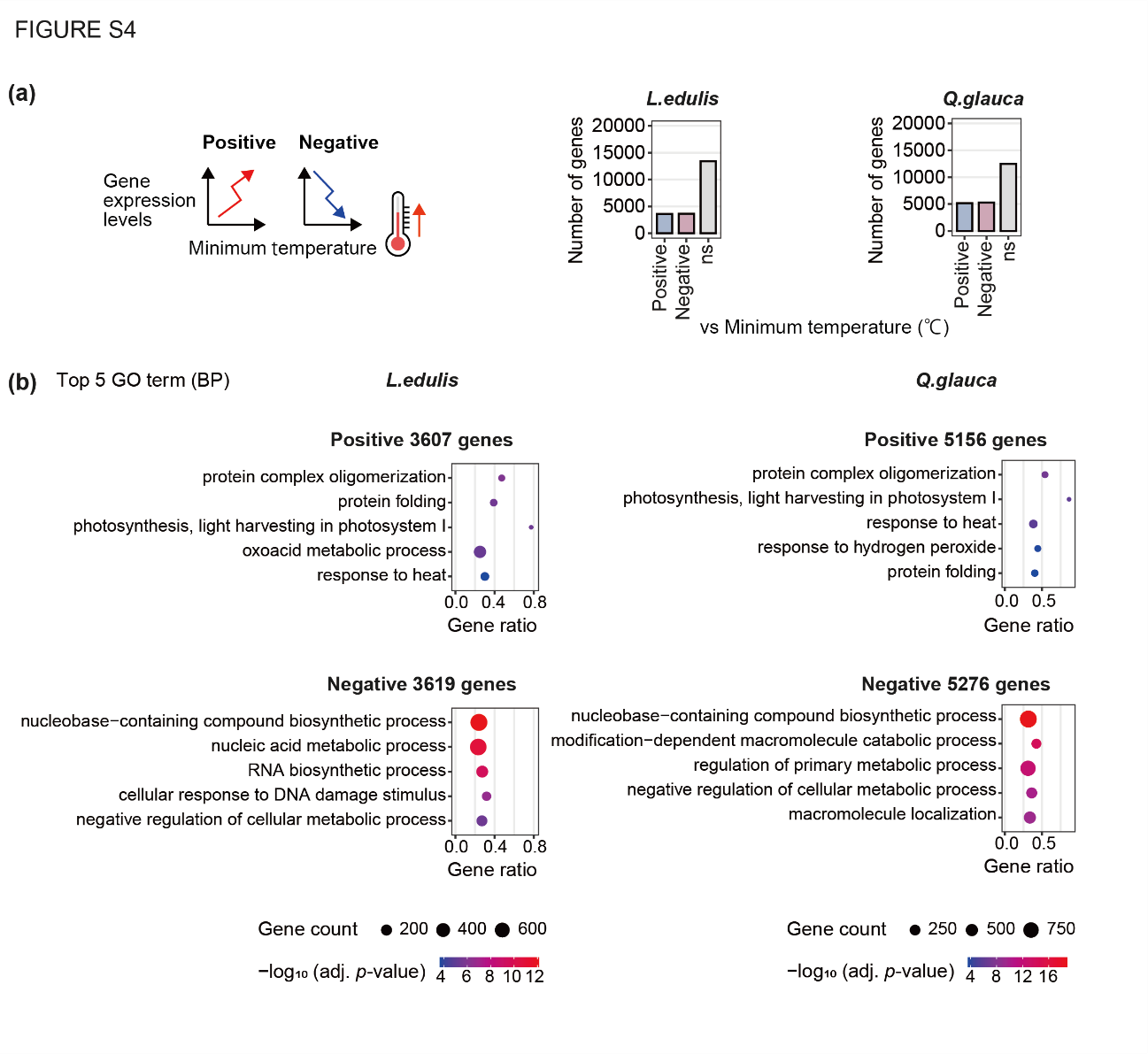


**FIGURE S4** Transcriptional responses to minimum temperature and GO enrichment analysis in two temperate species. (a) Number of genes showing transcriptional responses to minimum temperature in *L. edulis* and *Q. glauca*. The response type is classified as “Positive” (expression increases with higher temperature), “Negative” (expression decreases with higher temperature), or “ns” (no significant change). (b) GO enrichment analysis of genes significantly responsive to minimum temperature in *L. edulis* and *Q. glauca*, including 3607 *L. edulis* and 5156 *Q. glauca*, genes with increased expression (“Positive”) and 3619 *L. edulis* and 5276 *Q. glauca*, genes with decreased expression (“Negative”). The plot displays the top 5 significantly enriched Gene Ontology (GO) terms related to biological processes. The size of each dot reflects the gene count, and the colour scale represents the adjusted *p*-value for enrichment, with blighter colours indicating higher statistical significance. Gene ratio was calculated as the number of significantly responsive genes associated with each GO term divided by the total number of genes annotated to that term.

**Table S1.** Sample information for the tropical tree species *R. leprosula*.

**Table S2.** Orthogroup list of expressed genes of *R. leprosula*, *L. edulis*, and *Q. glauca* used in Figure 2.

**Table S3.** List of temperature-responsive genes of *R. leprosula*, *L. edulis*, and *Q. glauca* shown in Figure 3a and S4a, respectively.

**Table S4.** Top 30 GO terms for temperature-responsive genes of *R. leprosula*, *L. edulis*, and *Q.glauca* shown in Figure 3b and S4b, respectively.

**Table S5.** Gene list of genes related to photosynthesis and stress responses presented in Figure 3c.

**Table S6.** Temperature-responsive genes (577 absolute, 239 normalized temperature) among the 3793 single-copy orthologous genes used to compare temperature sensitivity in response to minimum temperature between tropical and temperate species in Figure 4.

**Table S7.** List of 228 temperature-responsive genes identified across tropical and temperate species shown in Figure 4.
